# A Comprehensive Review on the Medicinal Plants from the Genus *Asphodelus*

**DOI:** 10.3390/plants7010020

**Published:** 2018-03-13

**Authors:** Maryam Malmir, Rita Serrano, Manuela Caniça, Beatriz Silva-Lima, Olga Silva

**Affiliations:** 1 Research Institute for Medicines (iMed.ULisboa), Faculty of Pharmacy, Universidade de Lisboa, Av. Professor Gama Pinto, 1649-003 Lisbon, Portugal; mmalmir@ff.ulisboa.pt (M.M.); rserrano@ff.ulisboa.pt (R.S.); beatrizlima@netcabo.pt (B.S.-L.); 2 Department of Infectious Diseases, National Reference Laboratory of Antibiotic Resistances and Healthcare Associated Infections, National Institute of Health Doutor Ricardo Jorge, 1649-016 Lisbon, Portugal; Manuela.Canica@insa.min-saude.pt

**Keywords:** anthracene derivatives, antimicrobial, *Asphodelus*, ethnomedicine, skin diseases

## Abstract

Plant-based systems continue to play an essential role in healthcare, and their use by different cultures has been extensively documented. *Asphodelus* L. (*Asphodelaceae*) is a genus of 18 species and of a total of 27 species, sub-species and varieties, distributed along the Mediterranean basin, and has been traditionally used for treating several diseases particularly associated with inflammatory and infectious skin disorders. The present study aimed to provide a general review of the available literature on ethnomedical, phytochemical, and biological data related to the genus *Asphodelus* as a potential source of new compounds with biological activity. Considering phytochemical studies, 1,8-dihydroxyanthracene derivatives, flavonoids, phenolic acids and triterpenoids were the main classes of compounds identified in roots, leaf and seeds which were correlated with their biological activities as anti-microbial, anti-fungal, anti-parasitic, cytotoxic, anti-inflammatory or antioxidant agents.

## 1. Introduction

The genus *Asphodelus* Linnaeus belongs to family *Asphodelaceae* Jussieu and is native to temperate Europe, the Mediterranean, Africa, the Middle East, and the Indian Subcontinent, and now naturalized in other places (New Zealand, Australia, Mexico, southwestern United States, etc.) [1]. It reaches its maximum diversity in the West of the Mediterranean, particularly in the Iberian Peninsula and in North-West Africa [2].

The family consists of three subfamilies: *Asphodeloideae* Burnett (including 13 genera), *Hemerocallidoideae* Lindley (including 19 genera) and *Xanthorrhoeoideae* M.W. Chase (with only one genus). This botanical family, now called *Asphodelaceae*, has had a complex history; its circumscription and placement in an order have varied widely. In the Cronquist system of 1981, members of the *Asphodelaceae* were placed in the order Liliales Perleb [3,4]. Cronquist had difficulty classifying the less obviously delineated lilioid monocots; consequently, he placed taxa from both the modern orders Asparagales Link and Liliales into a single family, *Liliaceae* Jussieu [5]. The decision to group three formerly separate families, *Asphodelaceae*, *Hemerocallidaceae* and *Xanthorrhoeaceae*, into a single family first occurred in 2003 as an option in the II Angiosperm Phylogeny Group (APG) classification for the orders and families of flowering plants. The name used for the broader family was then *Xanthorrhoeaceae* Dumortier [6], and the earlier references to this family were related only to subfamily *Xanthorrhoeoideae*. These changes were a consequence of improvements in molecular and morphological analysis and also a reflection of the increased emphasis on placing families within an appropriate order [5,7,8]. Later in 2009, the APG III classification dropped the option of keeping the three families separate, using only the expanded family, still under the name *Xanthorrhoeaceae* [7]. Anticipating a decision to conserve the name *Asphodelaceae* over *Xanthorrhoeaceae*, the APG IV classification of 2016 used *Asphodelaceae* as the name for the expanded family [9].

According to the World Checklist of Selected Plant Families (WCSP), there are 32 accepted names with more than 150 homo- and heterotypic synonyms for all species, subspecies and varieties of the genus *Asphodelus* L. namely, *Asphodelus acaulis* Desfontaines, *Asphodelus aestivus* Brotero, *Asphodelus albus* Miller (subsp. *albus*; subsp. c*arpetanus* Z. Díaz & Valdés; subsp. *delphinensis* (Grenier & Godron) Z. Díaz & Valdés; subsp. *occidentalis* (Jordan) Z. Díaz & Valdés), *Asphodelus ayardii* Jahandiez & Maire, *Asphodelus bakeri* Breistroffer, *Asphodelus bento-rainhae* P. Silva (subsp. *bento-rainhae*; subsp. *salmanticus* Z. Díaz & Valdés), *Asphodelus cerasiferus* J. Gay, *Asphodelus fistulosus* Linnaeus (subsp. *fistulosus*; subsp. *madeirensis* Simon), *Asphodelus gracilis* Braun-Blanquet & Maire, *Asphodelus lusitanicus* Coutinho (var. *lusitanicus*; var. *ovoideus* (Merino) Z. Díaz & Valdés), *Asphodelus macrocarpus* Parlatore (subsp. *macrocarpus*; subsp. *rubescens* Z. Díaz & Valdés; var. *arrondeaui* (J. Lloyd) Z. Díaz & Valdés), *Asphodelus ramosus* Linnaeus (subsp. *distalis* Z. Díaz & Valdés; subsp. *Ramosus*); *Asphodelus refractus* Boissier, *Asphodelus roseus* Humbert & Maire, *Asphodelus serotinus* Wolley-Dod, *Asphodelus tenuifolius* Cavanilles, and *Asphodelus viscidulus* Boissier [1]. However, on the Missouri Botanical Garden database (Tropicos), more two accepted names (*Asphodelus cerasifer* Gay and *Asphodelus microcarpus* Salzmann & Viviani) were recorded [10]. Considering all the above-mentioned data 18 species and of a total of 27 species, sub-species and varieties must be considered for the *Asphodelus* genus.

Among all the species, *A. aestivus* and *A. fistulosus* are inscribed as “Least Concern” and *A. bento-rainhae* as “Vulnerable” species on International Union for the Conservation of Nature (IUCN) Red List of Threatened Species [11].

Botanical and systematic descriptions of this genus have been discussed by several taxonomists in various flora publications. The plants are hardy herbaceous perennials with narrow tufted radical leaves and an elongated stem bearing a spike of white or yellow flowers. Many have a small rhizomatous crown and thick, fleshy roots [12].

Different ethnomedical uses were described to *Asphodelus* species. Different parts of the plant including leaf, fruit, seed, flower, and root are used as traditional herbal medicines, alone or in mixtures to treat various ailments. In Iberian Peninsula, the following general medicinal uses were described: by rubbing with the cut tubers for the treatment of skin eczema, the ashes of the roots were used against the alopecia, and the leaves and stems decoction was used for the treatment of paralysis and the juice of fresh capsules for earache treatment [2]. Medicinal usage of the *Asphodelus* genus is also common in North African, and West and South Asian countries. Beside its medicinal uses, in Iberian Peninsula the alcohol obtained by fermentation of the tubers is extracted and used as fuel [2] and the local people of Iran, Turkey and Egypt use the root tubers of *A. aestivus* and *A. microcarpus* to produce a strong glue used by shoemakers and cobblers [2,13,14], and as yellow and brown dyes to dye the wool [2].

Root tubers are used as daily food, after being moistened and fried beforehand to eliminate the astringent compounds, and also the young stem, the leaves and the roasted seeds [2,15].

This study aims to present a comprehensive and updated review of documented ethnomedicinal and ethnopharmacological studies including chemical and biological data concerning *Asphodelus* genus.

## 2. Results and Discussion

Table 1 summarizes the ethnomedicinal data about the *Asphodelus* species including specific information on the plant parts as well as the geographical region where the plant is used. In Table 2 the principal chemical studies and identified compounds of the genus are presented. Table 3 and Table 4 summarize the principals of in vitro and in vivo biological activity assays on the total extracts and isolated compounds.

### 2.1. Ethnomedical Studies

Ethnomedicinal records showed that among the 18 species of the genus *Asphodelus,* only five species namely *A. aestivus*, *A. fistulosus*, *A. microcarpus*, *A. ramosus*, and *A. tenuifolius* have been documented for their traditional uses (Table 1). Most commonly, these species were used as anti-inflammatory and anti-infective agents. In particular, *A. aestivus*, *A. fistulosus* and *A. microcarpus* were reported to be used in dermatomucosal infections in various countries including Cyprus, Egypt, Libya, Palestine, and Spain [16,17,18,19,20]. *A. microcarpus*, *A. ramosus* and *A. tenuifolius* were generally indicted as anti-inflammatory agents specifically for the treatment of psoriasis, eczema, and rheumatism [21,22,23,24,25,26,27,28]. *A. aestivus* and *A. tenuifolius* are also used for ulcer treatment in Turkey, India, and Pakistan [26,27,28,29]. *A. ramosus* and *A. tenuifolius* have frequently been reported as diuretics among the inhabitants of Egypt, India, Pakistan, and Turkey [24,25,27,28,29,30,31].

### 2.2. Phytochemical Studies

Phytochemical studies as shown in Table 2, revealed the presence of different groups of compounds namely anthraquinones (either in the free or in the glycoside form), phenolic acids, flavonoids, and triterpenoids from *A. acaulis*, *A. albus*, *A. aestivus*, *A. cerasiferus*, *A. fistulosus*, *A. microcarpus*, *A. ramosus*, and *A. tenuifolius.*

Roots were mainly reported to have anthraquinone derivatives such as chrysophanol and aloe-emodin, triterpenoids, and naphthalene derivatives, while aerial parts mostly exhibited the presence of flavonoids such as luteolin, isovitexin and isoorientin, phenolic acids, and few anthraquinones. Fatty acids, namely myristic, palmitic, oleic, linoleic, and linolenic, were found in seeds and roots. Only *A. aestivus* and *A. microcarpus* were studied for essential oil characterization of flowers [22,35].

### 2.3. Reported Biological Activities

In vitro and in vivo biological studies concerning *Asphodelus* extracts are presented in Table 3 and those reported from identified pure compounds are shown in Table 4. In some of the studies, no data were obtained concerning the tested doses and/or inhibitory values.

The ethanol and aqueous extracts of *A. aestivus* leaf showed moderate anti-fungal activity against *Aspergillus niger* [33], and whole plant ethanol extracts exhibited weak activity against *Staphylococcus aureus* with minimum inhibitory concentration (MIC) = 42 mg/mL) and *Klebsiella pneumoniae* (MIC = 60 mg/mL) [67]. Both leaf and root extracts showed strong antioxidant activity [15,68]. The root extract also showed significant anti-inflammatory properties, specifically anti-ulcer activity which is one of the documented uses in Turkish traditional medicine [32]. Root and leaf extracts showed antitumoral activity against human cancer cells (lung and prostate) through DNA damage [68,69].

The aerial parts extracts of *A. luteus* showed strong anti-fungal activity against *Trichophyton violaceum* (MIC = 18 µg/mL), *Microsporum canis* (MIC = 25 µg/mL), and *Trichophyton mentagrophytes* (MIC = 30 µg/mL) supporting their traditional use in dermatomucosal infections [18] and weak activity against methicillin-resistant *Staphylococcus aureus* (MRSA) isolates (MIC = 1.25–2.5 mg/mL) [70]. Moreover, the methanol root extracts showed moderate antioxidant activity against 2,2-diphenyl-1-picrylhydrazyl free radicals (DPPH; IC_50_ = 0.54 mg/mL) [61].

The aerial parts and root extracts of *A. microcarpus* showed moderate antioxidant [47,61] and moderate to weak cytotoxic activities [48,49,71]. The ethanol extracts of leaves demonstrated strong antiviral activity against Ebola virus (EBOV) in the concentration of 0.1–0.3 µg/mL [49]. Although the leaf seems to have stronger antimicrobial activity in comparison with roots, in general, both exhibit weak or no antimicrobial/antifungal activity [20,48,49,70]; however, compounds isolated from root tubers extracts showed potent activity such as asphodelin A against **S. aureus** (MIC = 16 µg/mL), *Escherichia coli* (MIC = 4 µg/mL), *Pseudomonas aeruginosa* (MIC = 8 µg/mL), *Candida albicans* (MIC = 64 µg/mL) [19] and *Botrytis cinerea* (MIC = 128 µg/mL) and asphodoside B against MRSA (IC_50_ = 1.62 μg/mL) [51]. Other isolated compounds from root extracts showed different biological activity; for instance, ramosin showed potent cytotoxic activity against leukemia cell lines [21], aestivin showed potent antimalarial activity against chloroquine-sensitive and resistant strains of *Plasmodium falciparum* with IC_50_ of 0.8–0.7 μg/mL [21] and 3,4-dihydroxy-methyl benzoate exhibited anti-parasitic activity against *Leishmania donovani* promastigotes with IC_50_ of 33.2 µg/mL [54].

Root extracts of *A. ramosus* showed positive in vivo anti-inflammatory activity, confirming the traditional uses of the plant in inflammatory disorders [23].

Several root, seed, aerial parts, fruit, and leaf extracts of *A. tenuifolius* showed strong anti-microbial/antifungal against *K. pneumoniae*, *P. aeruginosa*, *E. coli*, *S. aureus*, *Proteus mirabilis*, *C. albicans*, *Aspergillus fumigatus*, *Vibrio cholerae*, *Salmonella typhi*, and *Candida glabrata*, among other pathogens [24,27,30,34,72,73]. Of note, there is no ethnomedical report of antimicrobial use of *A. tenuifolius*.

The whole plant extract showed in vivo hypotensive and diuretic activity in normotensive rats [74]. The root extract of this species showed anti-oxidant activity (DPPH test, IC_50_ = 2.006 µg/mL) [25] and asphorodin, a compound isolated from the whole plant extract, exhibit a potent inhibition of lipoxygenase enzyme, (IC_50_ = 18.1 µM) [26], which may have an important role as an anti-inflammatory agent. The biological properties of *A. tenuifolius* extracts prove their ethnomedical use mostly as anti-inflammatory or diuretic [24,25,26,27,28,30,31,34].

## 3. Materials and Methods

Ethnobotanical data was collected by our team in Portugal and relevant literature was reviewed until December 2017, by probing scientific databases (PubMed, Scopus, Google Scholar, b-on, Web of knowledge) and other web sources such as records from WCSP, IUCN, APG and the Missouri Botanical Garden database. Various keywords were used during the bibliographic research including: *ASPHODELUS* SPECIES; TRADITIONAL USES; ETHNOMEDICINAL EVIDENCE; BIOLOGICAL ACTIVITIES; ISOLATED MOLECULES; PHYTOCHEMISTRY. Information was gathered and summarized in table form where appropriate.

## 4. Conclusions

In conclusion, among the 18 species of the genus *Asphodelus,* only 30 percent of the species namely *A. aestivus*, *A. fistulosus*, *A. microcarpus*, *A. ramosus*, and *A. tenuifolius* have been documented for their traditional uses. In phytochemical studies 50 percent of the species (*A. acaulis*, *A. aestivus*, *A. albus*, *A. cerasifer*, *A. fistulosus*, *A. macrocarpus*, *A. microcarpus*, *A. ramosus*, *A. tenuifolius*) have been evaluated for their constituents however there is no documented data related to traditional uses of *A. acaulis*, *A. albus* and *A. cerasiferus.*

All the species with ethnomedical documented data were submitted to biological activity tests, showing a total or partial correlation with their traditional use as anti-microbial, anti-fungal, anti-parasitic, cytotoxic, anti-inflammatory, or antioxidant agents.

Root tubers plant part were mainly reported to have anthraquinone derivatives, triterpenoids, and naphthalene derivatives, while aerial parts mostly exhibited the presence of flavonoids, phenolic acids, and few anthraquinones.

Considering the previous phytochemical studies, 1,8 dihydroxyanthracene derivatives (e.g., aloe-emodin and chrysophanol) were the most common reported anthraquinones of *A. aestivus*, *A. luteus* and *A. microcarpus* extracts which could be responsible for the reported antimicrobial/fungal activities [78,80]. Aloe-emodin as a potent cytotoxic compound might be related to the reported anti-tumoral activity of *A. aestivus* [68,78]*.*

Flavonoids namely luteolin and apigenin derivatives were frequently reported from the aerial parts of all studied *Aphodelus* species, which according to their known antioxidant and anti-inflammatory properties [81,82], could be correlated to their traditional uses in inflammatory diseases in agreement with the reported biological studies. Phenolic acids, namely caffeic acid and chlorogenic acid reported from aerial parts and root tubers might be responsible for the general antioxidant activity presented in the biological studies.

Phytosterols (e.g., fucosterol, β-sitosterol, and stigmasterol) and β-amyrin were the most common found triterpenoids from roots and seeds. According to the literature, β-amyrin possess antibacterial/antifungal properties [83] which complement the reported biological activities of *A. tenuifolius*.

The present study allowed the importance and potential of the genus *Asphodelus* as a source of new compounds to be ascertained, with biological activity and new herbal products based on *Asphodelus* genus used in traditional medicine being ascertained, as well as its quality, mode of action, and safety of use. It should be pointed out that, to the best of our knowledge, the latter aspect (the safety of *Asphodelus* species) has not yet been the object of in-depth studies.

## Figures and Tables

**Table 1:** Ethnomedicinal uses of the *Asphodelus* species.

Species	Part Used	Country	Traditional Uses/Application	References
***A. aestivus***	L, R	Turkey	Peptic ulcers	[32]
	R	Turkey	Haemorrhoids, burns, wounds and nephritis	[33]
	NI	Cyprus, Spain	Skin diseases	[16]
***A. fistulosus***	NI	Egypt, Libya	Fungal infections	[17]
A. luteus *	WP	Palestine	Dermatomucosal infections	[18]
***A. microcarpus***	FR, L, R	Egypt	Ear-ache, withering and paralysis	[13,14]
	R	Palestine	Dermatomucosal infections	[18]
	R	Egypt	Ectodermal parasites, jaundice, microbial infections and psoriasis	[19,20,21]
	NI	Algeria	Ear-ache, eczema, colds and rheumatism	[22]
***A. ramosus***	R	North-Africa	Inflammatory disorders	[23]
	NI	Turkey	Anti-tumoral, diuretic and emmenagogue	[29]
***A. tenuifolius***	L	India	Diuretic, inflammatory disorders and ulcers	[24]
	L, SE	Egypt	Diuretic	[30]
	R, SE	India	Antipyretic, diuretic, colds and hemorrhoids, inflammatory disorders, rheumatic pain, ulcers and wounds	[25,27]
	SE	Pakistan	ulcers and inflammatory disorders	[26]
	WP	India	Diuretic, inflammatory disorders, bite of bees and wasps, ulcers	[28,34]
	NI	Pakistan	Diuretic	[31]

SE: Seed; L: Leaf; WP: Whole plant; FR: Fruit; R: Root; NI: Not indicated, * *Asphodelus luteus* L.—synonym of *Asphodeline lutea* was formerly included in the family *Asphodelaceae.*

**Table 2:** Identified compounds reported from *Asphodelus* genus.

Species	Part Used	Class	Name of Compounds	References
***A. acaulis***	L	Flavonoids	Luteolin; apigenin	[36]
	R	Anthraquinones	Chrysophanol; asphodelin; 10,7′-bichrysophanol	[37]
***A. aestivus***	FL	*n*-alkenes	Hexadecanoic acid (35.6%), pentacosane (17.4%), tricosane (13.4%), heptacosane (8.4%), heneicosane (4.5%), phytol (4.5%), tetracosane (3%), hexacosane (2%), hexahydrofarnesyl acetone (1.7%), tetradecanoic acid (1.4%), docosane (1.3%), nonadecane (1%)	[35]
	L	Amino acids	Adenosine; tryptophan; phenylalanine	[38]
		Anthraquinones	Aloe-emodin; aloe-emodin acetate; chyrosphanol 1-*O*-gentiobioside	
		Flavonoids	Isovitexin; isoorientin; isoorientin 4′-*O*-β glucopyranoside; 6′′-*O*-(malonyl)-isoorientin; 6′′-*O*-[(*S*)-3-hydroxy-3-methylglutaroyl]-isoorientin	
		Phenolic acid	Chlorogenic acid	
	SE	Fatty acids	Butyric acid; nervoic acid	[39]
***A. albus***	L	Anthraquinones	Aloe-emodin; chrysophanol	[36,40]
		Flavonoid	Luteolin	[36]
	R	Anthraquinones	Chrysophanol; asphodelin; 10,7′-bichrysophanol	[37]
		Fatty acids	Myristic (5.3%); palmitic (18.5%); stearic (2.1%); oleic (13.5%); linoleic (44.1%); linolenic (9.9%); arachidic (2.7%); behenic (1.2%); lignoceric (2.1%) acids	[41]
		Triterpenoids	β-sitosterol; β-amyrin; campesterol; stigmasterol; fucosterol	
***A. albus* var. *delphinensis***	R	Anthraquinones	Asphodeline; microcorpine; aloe-emodine; chrysophanole	[42]
***A. cerasifer***	L	Anthraquinones	Aloe-emodin	[36]
		Flavonoids	Isoorientin; luteolin; luteolin 7-glucoside	[36,43]
	R	Anthraquinones	Asphodeline; microcorpine; aloe-emodine; chrysophanole	[41]
* A. delphinensis	L	Flavonoids	Isoorientin; luteolin; luteolin 7-glucoside	[43]
***A. fistulosus***	AP	Anthraquinones	Asphodelin; asphodelin 10′-anthrone; aloesaponarin II; aloe-emodin; chrysophanol; desoxyerythrolaccin	[17]
		Flavonoids	Chrysoeriol; luteolin	
	L	Anthraquinones	Dianhydrorugulosin; aloe-emodin; chrysophanol; 1,8 hydroxy-dianthraquinone	[44]
	R	Anthraquinones	Chrysophanol; asphodelin; 10,7′-Bichrysophanol	[37]
	SE	Anthraquinones	Dianhydrorugulosin; aloe-emodin; chrysophanol; 1,8 hydroxy-dianthraquinone	[44]
		Carbohydrates	Sucrose; raffinose; stachyose	[45]
		Fatty acids	Myristic (0.5%); palmitic (5.7%); stearic (3.6%); oleic (33.1%); linoleic (54.9%)	[45,46]
		Triterpenoids	β-sitosterol; β-amyrin	[45]
** A. luteus	L	Anthraquinones	Aloe-emodin	[36]
*** A. mauritii Sennen	L	Anthraquinones	Aloe-emodin; chrysophanol	[36]
		Flavonoids	Luteolin	
***A. microcarpus***	FL	Terpenoids	Germacrene D (78.3%); germacrene B (3.9%); a-elemene (3.8%); caryophyllene (3.3%)	[22]
		Flavonoids	Luteolin; luteolin-6-*C*-glucoside; luteolin-*O*-hexoside; luteolin-7-*O*-glucoside; luteolin-*O*-acetylglucoside; luteolin-*O*-deoxyhesosylhexoside; methyl-luteolin, naringenin; apigenin	[47]
		Phenolic acids	3-O caffeoylquinic acid; 5-O caffeoylquinic acid	
	L	Anthraquinone	Chrysophanol, 10 (chrysophanol-7-yl)-10-Hydroxychrysophanol-9-antrone, asphodoside C, Dianhydrorugulosin; aloe-emodin	[44,48]
		Flavonoids	Luteolin-6-*C*-glucoside; luteolin-6-c-acetilglucoside; luteolin-*C*-glucoside; luteolin, isoorientin	[43,49]
		Phenolic acids	5-O caffeoylquinic acid; cichoric acid; cumaril exosa malic acid	[49]
	R	Anthraquinones	Dianhydrorugulosin; aloe-emodin; chrysophanol; asphodelin; microcarpin, 8 methoxychrysophanol; emodin; 10-(chrysophanol-7′-yl)-10-hydroxychrysophanol-9-anthrone; aloesaponol-III-8-methyl ether; ramosin; aestivin, asphodosides A-E, chrysophanol dianthraquinone; 5,5′-bichrysophanol; chrysophanol-8-mono-β-d-glucoside; Methyl-1,4,5-trihydroxy-7-methyl-9,10-dioxo-9,10-dihydroanthracene-2-carboxylate; 6 methoxychrysophanol	[21,44,50,51,52,53,54]
		Arylcoumarins	Asphodelin A 4′-*O*-β-d-glucoside; asphodelin A	[19]
		Carbohydrates	Raffinose; sucrose; glucose; fructose	[55]
		Fatty acids	Palmitic; stearic; oleic; linoleic; linolenic; arachidic; behenic; lignoceric; myristic acids	[55,56]
		Naphthalene derivatives	2-acetyl-1,8-dimethoxy-3 methylnaphthalene; 1,6-dimethoxy-3-methyl-2-naphthoic acid	[21]
		Mucilage	Composed of glucose; galactose; arabinose	[55]
		Triterpenoids	β-sitosterol-β-d-glucoside, fucosterol	[13,55]
	SE	Anthraquinones	Aloe-emodin; chrysophanol; chrysophanol-8-mono-β-d-glucoside	[44]
		Carbohydrates	Sucrose; raffinose; stachyose; melibiose	[45]
		Fatty acids	Myristic; palmitic; stearic; oleic; linoleic acids	
		Triterpenoids	β-sitosterol; β-amyrin	
***A. ramosus***	FL	Flavonoids	Luteolin	[57]
		Phenolic acids	Caffeic acid; chlorogenic acid; *p*-hydroxy-benzoic acids	
	L	Flavonoids	Luteolin; 7-*O*-glucosyl luteolin; 7-*O*-glucosyl apigenin; isoorientin; isoswertiajaponin (7-methyl orientin); isocytisoside (4′-methyl vitexin)	[29]
	R	Anthraquinone	Ramosin; (−)-10′-*C*-[β-d-xylopyranosyl]-; (−)-10′-*C*-[β-d-glucopyranosyl-(1-4)-β-d-glucopyranosyl]-1,1′,8,8,10,10′-hexa hydroxy -3,3′-dimethyl-10,7′ bianthracene-9,9′-dione; 10′-deoxy-10-epi-ramosin; 10-(chrysophanol-7′-yl)-10-hydroxychrysophanol-9-anthrone; 7′-(Chrysophanol-4-yl)-chrysophanol-10′anthrone10′-*C*-α-rhamnopyranosyl; -*C*-β-xylopyranosyl; -*C*-β-antiaropyranosyl; -*C*-α-arabinopyranosyl; -*C*-β-quinovoopyranosyl	[58,59,60]
	WP	Flavonoids	Naringin, quercetin, kaemferol	[61]
		Phenolic acids	Gallic acid, chlorogenic acid, vanilic acid, cafeic acid	
***A. tenuifolius***	AP	Flavonoids	Luteolin; luteolin-7-*O*-β-d-glycopyranoside; apigenin, chrysoeriol	[30]
	R	Naphthalene derivatives	1,8-dimethylnaphthalene; 2-acetyl-8-methoxy-3-methyl-1-naphthol; 2-acetyl-1,8-dimethoxy-3-methylnaphthalene	[62]
		Triterpenoids	β-sitosterol; stigmasterol	
	SE	Ester	1-*O*-17methylstearylmyoinositol	[63]
		Fatty acids	Myristic (3.96%); palmitic (13.84%); oleic (15.60%); linoleic (62.62%); linolenic (2.60%)	[64,65]
	WP	Amino acids	Crystine; serine; glycine; proline; alanine, glycin; serine; alanine and valine in the form of protein	[66]
		Carbohydrates	d-glucose; lactose; d-glucuronic acid; d-arabinose; d-fructose, d-ribose	
		Chromone	2-hentriacontyl-5,7-dihydroxy-8-methyl-4*H*-1-benzopyran-4-one	[31]
		Triterpenoids	Asphorodin; asphorin A; asphorin B; β-sitosterol; β-amyrin	[26,28,31]

AP: Aerial Part; FL: Flower; FR: Fruit; L: Leaf; R: Root; SE: Seed; WP: Whole plant; NI: Not indicated; * The accepted name is *Asphodelus albus* subsp. *delphinensis* (Gren. & Godr.). ** *Asphodelus luteus* L.—synonym of *Asphodeline lutea* was formerly included in the family *Asphodelaceae*. *** The accepted name is *Asphodelus macrocarpus* subsp. *rubescens.*

**Table 3:** In vitro and in vivo biological studies reported from the *Asphodelus* genus.

Species	Part	Extract	Test/Assay	Result	Reference
***A. aestivus***	L	Aqueous, Ethanol	In vitro anti-fungal activity (**A. niger**)—Agar well diffusion method (zone of inhibition in cm^−1^)	Ethanol extract (0.25 and 0.5 mg/mL) showed higher activity than aqueous extract (0.25 and 0.5 mg/mL) and similar activity for concentrations of 1 mg/mL. Both extracts were less active than Fluconazole (100 µ/mL)	[33]
			In vitro antioxidant activity—β-carotene bleaching effect, metal chelating, total antioxidant activity, DPPH, ABTS, superoxide radical scavenging activity, hydroxyl radical scavenging activity, DMPD, nitric oxide scavenging activity	Aqueous extract presented higher activity in metal chelating and radical scavenging assays (DPPH, IC_50 aqueous_ = 4.58 mg/mL and IC_50 methanol_ = 9.54 mg/mL, superoxide, hydroxyl, DMPD) Ethanol extract presented higher activity in β-carotene bleaching effect and total antioxidant activity Aqueous and ethanolic extracts presented similar radical scavenging activity in ABTS and NO assays. Both extracts presented significantly inferior results when compared to reference substances	
***A. aestivus***	L	Acetone, Methanol	In vitro antioxidant activity—β-carotene, reducing power assay, DPPH, ABTS, inhibition of linoleic acid peroxidation, superoxide radical scavenging assays	Reducing power and total antioxidant activity were higher in acetone extract; free radical and superoxide radical scavenging activity were higher in methanol extract (DPPH, IC_50 methanol_ = 0.16 mg/mL and IC_50 acetone_ = 0.50 mg/mL) Acetone extract presented higher activity in Reducing power and total antioxidant activity (inhibition of linoleic acid peroxidation) Methanol extract presented higher activity in superoxide radical scavenging and free radical scavenging activity (β-carotene, ABTS and DPPH, IC_50 methanol_ =0.16 mg/mL, IC_50acetone_ = 0.50 mg/mL)	[15]
***A. aestivus***	L, R	Dichloromethane *n*-Hexane	In vitro cytotoxic activity—MTT assay against human lung cell cancer (A549) and prostate cell cancer (PC3)	Root: Dichloromethane: A549 (IC_50_ = 16 µg/mL); PC3 (IC_50_ = 19 µg/mL) *n*-Hexane: PC3 (IC_50_ = 80 µg/mL) Leaves: Dichloromethane: A549 (IC_50_ = 90 µg/mL)	[69]
***A. aestivus***	R	Aqueous (decoction)	In vivo anti-inflammatory—Ethanol induced gastric ulcer model in rats	Decoction gave significant protection against the lesions	[32]
***A. aestivus***	R	Aqueous (infusion and decoction) Diethyl ether, Ethyl acetate, Methanol	In vitro antioxidant activity—DPPH assay	Diethyl ether (IC_50_ = 22.46 µg/mL) have a higher scavenging activity than Ethyl acetate (IC_50_ = 188.90 µg/mL), both have lower activity than reference substance, rutin (7.77 µg/mL). Methanol and aqueous extract had no scavenging activity	[68]
			In vitro cytotoxic & apoptotic activity—MCF-7 breast cancer cells-trypan blue exclusion assay, comet assay, Hoechst 33,258, propidium iodide double staining	Methanol and aqueous extracts exhibited strong cytotoxic activities. All extracts showed significant DNA damaging and apoptotic activities.	
***A. aestivus***	SE	Petroleum ether	In vitro antimicrobial/fungal activity—broth microdilution method	Active against *S. aureus* (MIC = 512 µg/mL), *Enteroococcus faecalis* (MIC = 512 µg/mL), *K. pneumoniae* (MIC = 512 µg/mL) and *C. albicans* (MIC = 512 µg/mL) Not active against *Bacillus cereus*, *Staphylococcus epidermidis*, *E. coli*, *P. aeruginosa*, *S. typhimurium*, *Salmonella enterica*, *Candida krusei* and *Candida parapsilosis*	[39]
***A. aestivus***	WP	*n*-Butanol, Ethanol	In vitro anti-microbial/fungal activity—well and disk diffusion method	Active against *S. aureus* (MIC: 42 mg/mL), *K. pneumoniae* (MIC: 60 mg/mL), *E. coli* (MIC: 90 mg/mL), *C. albicans* (MIC: 90 mg/mL)	[67]
***A. aestivus***	WP	Aqueous	In vitro antioxidant activity—DPPH assay	Inhibition % = 62.5	[75]
***A. fistulosus var. tenuifolius***	NI	NI	In vitro anti-microbial/fungal activity	Positive to *S. aureus* and no activity against *E. coli*, Proteus vulgaris, *Salmonella* sp., *P. aeruginosa*, *C. albicans*	[76]
A. luteus *	AP	Aqueous	In vitro anti-fungal activity—Agar dilution method	Activity against *T. violaceum* (MIC = 18 µg/mL), *M. canis* (MIC = 25 µg/mL) and *T. mentagrophytes* (MIC = 30 µg/mL)	[18]
A. luteus *	AP R	Methanol, Petroleum Ether	In vitro anti-microbial activity—agar diffusion test; tetrazolium microplate assay (MIC)	Against MRSA isolates Methanol extract: MIC (AP) = 1.25–2.5 mg/mL MIC (R) = 0.65–1.25 mg/mL Petroleum ether extract: Root extract had higher activity than aerial part extract	[70]
A. luteus *	R	Methanol	In vitro antioxidant activity—DPPH assay	IC_50_ (methnol)= 0.54 mg/mL, IC_50_ (reference, BHT) = 0.017 mg/mL	[61]
***A. microcarpus***	AP	Aqueous	In vitro anti-fungal activity—Agar dilution method	Weak activity against *T. violaceum* (MIC = 25 µg/mL) and no activity against *M. canis* and *T. mentagrophytes*	[18]
***A. microcarpus***	AP R	Methanol	In vitro anti-microbial activity—agar diffusion test; tetrazolium microplate assay (MIC)	Against MRSA isolates Methanol extract: MIC (AP) = 1.25–5 mg/mL MIC (R) = 1.25–2.5 mg/mL	[70]
***A. microcarpus***	FL L R	Aqueous, Ethanol, Methanol	In vitro antimelanogenic activity—tyrosinase inhibition (mushroom tyrosinase assay and mouse melanoma cells viability), kojic acid as positive control	Antimelanogenic activity Ethanol extract (F) had the highest tyrosinase inhibition activity in mushroom assay and melanoma cell assay	[47]
			In vitro antioxidant activity—DPPH and ABTS (reference—Trolox)	Antioxidant activity DPPH (best activity) Ethanol extract (F): IC_50_ = 28.4 µg/mL Ethanol extract (L): IC_50_ = 55.9 µg/mL Trolox: IC_50_ = 3.2 µg/mL	
***A. microcarpus***	L	Ethanol	In vitro antimicrobial/fungal activity—micro broth dilution method	Active against *Bacillus clausii* (MIC = 250 µg/mL), *S. aureus* (MIC = 250 µg/mL), *Staphylococcus haemolyticus* (MIC = 250 µg/mL) and *E. coli* (MIC = 500 µg/mL). No activity against *Streptococcus* spp. and yeasts	[49]
			In vitro antiviral activity (IFN-β induction)—luciferase reporter gene assay	Antiviral activity Active against EBOV in concentration of 0.1–3 µg/mL	
			In vitro cytotoxicity-Cell viability of A549 cells, positive control (camptothecin)	Cytotoxicity IC_50_ (extract) > 100 µg/mL IC_50_ (camptothecin) = 0.54 µg/mL	
***A. microcarpus***	L	Methanol	In vitro antimicrobial/fungal—two-fold serial dilution technique	Antimicrobial activity Active against *S. aureus* (MIC = 78 µg/mL), *Bacillus subtilis* (MIC = 156 µg/mL), *Salmonella* spp. (MIC = 313 µg/mL), *E. coli* (MIC = 125 µg/mL), *Aspergillus flavus* (MIC = 125 µg/mL), *C. albicans* (MIC = 78 µg/mL)	[48]
			In vitro antiviral activity—CPE inhibition assay against HSV-1 and HAV-10	Antiviral activity Moderate activity against Hepatitis A virus (HAV-10) and no activity against Herpes Simplex Virus (HSV-1)	
			In vitro cytotoxicity—viability assay against human tumor cell lines of the lung (A-549), colon (HCT-116), breast (MCF-7) and prostate (PC3). Cisplatin as standard	Cytotoxicity Highest activity against human lung carcinoma cells (A-549), IC_50_ = 29.3 µg/mL	
***A. microcarpus***	R	Methnol	In vitro antioxidant activity—DPPH assay	IC_50_ (Methnol) = 0.30 mg/mL, IC_50_ (reference, BHT) = 0.017 mg/mL	[61]
***A. microcarpus***	R	Methanol	In vitro anti-microbial—Disk diffusion assay	No activity against *S. aureus*, *B. subtilis* and *E. coli*	[20]
***A. microcarpus***	WP	Aqueous, Ethanol	In vitro antioxidant activity—DPPH assay	Ethanol extract (100 µg/mL) with moderate activity (inhibition percentage—60.3%) higher than aqueous extract (100 µg/mL, inhibition percentage—49.5%)	[71]
			In vitro cytotoxic activity—Trypan blue technique for Ehrlich Ascites Carcinoma Cells (EACC)	Weak anti-cancer activity of both extracts	
***A. ramosus***	R	Aqueous, Chloroform, Ethanol, Methanol	In vivo anti-inflammatory—Arachidonic acid test (mouse ear oedema) Carrageenan test (sub-plantar oedema)	Arachidonic acid test: Positive activity from chloroform and ethanol extracts Carrageenan test: No activity was observed	[23]
***A. ramosus***	WP	Aqueous, Methanol, Methanol 50%	In vitro antioxidant activity—DPPH assay at 35 °C and 65 °C	Aqueous extract at 65 °C had the highest inhibition percentage	[77]
***A. tenuifolius***	AP	Butanol, Ethyl acetate, Methylene-chloride	In vitro anti-microbial/fungal activity—Disc diffusion method	All extracts showed antimicrobial activity, the methylene-chloride as the most active against *S. aureus* (MIC = 1.6 mg/mL), *E. faecalis* (MIC = 1.0 mg/mL), *E. coli* (MIC = 1.8 mg/mL) and *P. aeruginosa* (MIC = 0.15 mg/mL) All extracts showed antifungal activity against *C. albicans*, *C. parapsilosis*, *C. glabrata*, *C. krusei*.	[30]
***A. tenuifolius***	FR	Acetone, Aqueous, Benzene, Chloroform, Methanol, Petroleum ether	In vitro anti-microbial/fungal activity—Kirk-bauer disc diffusion method	Significant activity against *S. aureus* (acetone, MIC = 125 µg/mL); *S. epidermidis* (acetone, MIC = 125 µg/mL; chloroform and methanol, MIC = 250 µg/mL); *P. vulgaris* (methanol, MIC = 250 µg/mL; chloroform, MIC = 125 µg/mL), *P. mirabilis* (benzene, MIC = 125 µg/mL; acetone and methanol, MIC = 250 µg/mL; chloroform, MIC = 500 µg/mL) *E. coli* (acetone, chloroform and methanol, MIC = 125 µg/mL); *K. pneumoniae* (acetone and methanol, MIC = 125 µg/mL; chloroform and benzene, MIC = 500 µg/mL); *P. aeruginosa* (acetone, MIC = 250 µg/mL; chloroform, MIC = 500 µg/mL); *C. albicans* (acetone, MIC = 125 µg/mL); *A. fumigatus* (benzene and chloroform, MIC = 250 µg/mL; acetone, MIC = 500 µg/mL)	[27]
***A. tenuifolius***	L	Acetone, Methanol	In vitro anti-microbial/fungal activity—Agar disc diffusion method	Methanol extract positive against *S. aureus*, *B. cereus*, *Citrobacter freundii*, *Candida tropicalis* and acetone extract was positive against *K. pneumoniae*, *C. tropicalis* and *Cryptococcus luteolus*	[24]
***A. tenuifolius***	R	Methanol	In vitro antioxidant activity—DPPH, ABTS^+^, NO, OH, O_2_^−^, ONOO^−^ assays, Oxidative DNA damage	Positive activity, DPPH (IC_50_ = 2.006 µg/mL), ABTS·+ (IC_50_ = 156.94 µg/mL), NO (nd), OH (IC_50_ = 50.13 µg/mL), O_2_^−^ (IC_50_ = 425.92 µg/mL) and ONOO- (IC_50_ = 3.390 µg/mL), oxidative DNA damage: 1.85 µg/mL of extract prevented DNA damage.	[25]
***A. tenuifolius***	R	Benzene, Chloroform, Ethyl acetate, Methanol, Petroleum ether	In vitro anti-microbial/fungal activity—Disc diffusion method	All extracts were active against *B. subtilis*, *P. vulgaris*, *P. aeruginosa*, *Trichophyton rubrum*, *E. coli*, *K. pneumoniae*, *Shigella sonnei*, *S. aureus*, *C. albicans*, *A. niger* and *A. flavus*	[72]
***A. tenuifolius***	SE	Aqueous, Ethanol, Methanol, Petroleum ether	In vitro anti-microbial/fungal activity—modified Kirby Bauer disc diffusion method	Petroleum ether: no antibacterial activity Ethanol: activity against *P. aeruginosa*, *Vibrio cholerae* and *S. aureus* (MIC = 16 µg/mL); *P. mirabilis*, *S. typhi*, Shigella flexneri and Serratia marcescens (MIC = 32 µg/mL). Methanol: activity against *S. aureus* (MIC = 16 µg/mL); *V. cholerae,* *P. aeruginosa*, *S. typhi*, *S. flexneri* and S. marcescens (MIC = 16 µg/mL) Aqueous: activity against *V. cholerae*, *S. aureus*, *S. typhi* and *S. flexneri* (MIC = 32 µg/mL); *P. aeruginosa* and *P. mirabilis* (MIC = 16 µg/mL). No antifungal activity against *C. albicans* and *A. niger*	[34]
***A. tenuifolius***	WP	Methanol	In vitro antimicrobial/fungal activity—disk diffusion method In vitro anti-parasitic activity—trophozoites growth inhibition assay	Good activity against *E. coli* and moderate activity against *S. aureus*, *S. typhi*, *K. pneumoniae*, *P. aeruginosa*, *C. albicans* and *A. niger* Active against *Giardia lamblia* (IC_50_ = 219.82 µg/mL) and Entamoeba histolytica (IC_50_ = 344.62 µg/mL)	[73]
***A. tenuifolius***	WP	Aqueous	In vivo hypotensive activity—blood pressure (BP) measure after parenteral administration of aqueous extracts in rats. Acetylcholine and verapamil as positive controls in co administration with atropine	Hypotensive activity The extract decreased blood pressure in normotensive rats (35.2% decrease with 30 mg/Kg), similar to Verapamil. The response was independent from atropine effect	[74]
			In vivo diuretic activity—measure of rat urine output and urinary electrolytes. After 6 hr administration. Saline solution and furosemide as controls	Diuretic activity Significant increase in urinary volume and electrolytes excretion with 300 and 500 mg/Kg	

AP: Aerial Part; FL: Flower; FR: Fruit; L: Leaf; R: Root; SE: Seed; WP: Whole plant; NI: Not indicated; * *Asphodelus luteus* L.—synonym of *Asphodeline lutea* was formerly included in the family *Asphodelaceae.* ABTS^+^: 2,2′-azinobis-(3-ethylbenzothiazole-6-sulphonate) radical cation, DMPD: *N*,*N*-dimethyl-p-phenylenediamine dihydrochloride, DPPH: 2,2-diphenyl-1-picrylhydrazyl radical, NO: nitric oxide radical, O2^.–^: superoxide anion radical , ^·^OH: hydroxyl radical, ONOO^-^: Peroxynitrite radicals, EBOV: Ebola virus.

**Table 4:** In vitro and in vivo biological studies reported from pure compounds isolated from *Asphodelus* genus.

Species	Pure Compounds	Test/Assay	Result	Reference
***A. microcarpus***	Asphodelin A 4′-*O*-β-d-glucoside (1), Asphodelin A (2)	In vitro antimicrobial/fungal activity—micro dilution assay	*S. aureus* (MIC_1_ = 128 µg/mL, MIC_2_ = 16 µg/mL), *E. coli* (MIC_1_ = 128 µg/mL, MIC_2_ = 4 µg/mL), *P. aeruginosa* (MIC_1_ = 256 µg/mL, MIC_2_ = 8 µg/mL), *C. albicans* (MIC_1_ = 512 µg/mL, MIC_2_ = 64 µg/mL) and *B. cinerea* (MIC_1_ = 1024 µg/mL, MIC_2_ = 128 µg/mL	[19]
	3-methyl anthraline, chrysophanol, and aloe-emodine	Psoriasis	Positive (patent)	[78,79]
	1,6-dimethoxy-3-methyl-2-naphthoic acid (1), asphodelin (2), chrysophanol (3), 8 methoxychrysophanol (4), emodin (5), 2-acetyl-1,8-dimethoxy-3-methylnaphthalene (6), 10-(chrysophanol-7′-yl)-10-hydroxychrysophanol-9-anthrone (7), aloesaponol-III-8-methyl ether (8), ramosin (9), aestivin (10)	In vitro anti-parasitic activity	Compounds **3** and **4** showed moderate to weak against a culture of *L. donovani* promastigotes (IC_50_ = 14.3 and 35.1 μg/mL, respectively)	[21]
		In vitro cytotoxic activity-Human acute leukemia HL60 cells/human chronic leukemia 562 cells	Compounds **7** and **9** exhibited a potent cytotoxic activity against leukemia LH60 and K562 cell lines	
		In vitro antimalarial activity—chloroquine sensitive & resistant strains of Plasmodium falciparum (plasmodial LDH activity)	Compound **10** showed potent antimalarial activities against both chloroquine-sensitive and resistant strains of *P. falciparum* (IC_50_ = 0.8–0.7 μg/mL) without showing any cytotoxicity to mammalian cells	
		In vitro anti-microbial/fungal activity	Compound **4** exhibited moderate antifungal activity against Cryptococcus neoformans (IC_50_ = 15.0 μg/mL), compounds **5**, **7** and **10** showed good to potent activity against methicillin resistant *S. aureus* (MRSA) (IC_50_ = 6.6, 9.4 μg/mL and 1.4 μg/mL respectively). Compounds 5, 8 and 9 displayed good activity against *S. aureus* (IC_50_ = 3.2, 7.3 and 8.5 μg/mL, respectively)	
	Methyl-1,4,5-trihydroxy-7-methyl-9,10-dioxo-9,10-dihydroanthracene-2-carboxylate (1), (1*R*) 3,10-dimethoxy-5-methyl-1*H*-1,4 epoxybenzo[*h*]isochromene (2), 3,4-dihydroxy-methyl benzoate (3), 3,4-dihydroxybenzoic acid (4), 6 methoxychrysophanol (6)	In vitro anti-parasitic activity	Compound **3** showed activity against a culture of *L. donovani* promastigotes (IC_50_ = 33.2 µg/mL)	[54]
		In vitro anti-microbial/activity	Compound **1** showed a potent activity against methicillin resistant *S. aureus* (MRSA) and *S. aureus* (IC_50_: 1.5 and 1.2 µg/mL, Respectively)	
	5 Compounds, Asphodosides A–E	In vitro anti-microbial activity	Compounds **2**–**4** showed activity against methicillin resistant *S. aureus* (MRSA) (IC_50_: 1.62, 7.0 and 9.0 µg/mL, respectively). activity against *S. aureus* (non-MRSA), IC_50_ = 1.0, 3.4 and 2.2 µg/mL, respectively	[51]
***A. tenuifolius***	Asphorodin	In vitro anti-inflammatory-inhibition of lipoxigenase enzyme	Potent inhibitory activity (IC_50_ = 18.1 µM), Reference: baicalein (22.6 µM)	[26]
